# Antioxidative and Antimelanogenesis Effect of* Momordica charantia* Methanol Extract

**DOI:** 10.1155/2019/5091534

**Published:** 2019-05-02

**Authors:** Sang Hee Park, Young-Su Yi, Mi-Yeon Kim, Jae Youl Cho

**Affiliations:** ^1^Department of Biocosmetics, Sungkyunkwan University, Suwon 16419, Republic of Korea; ^2^Department of Pharmaceutical Engineering, Cheongju University, Cheongju 28503, Republic of Korea; ^3^School of Systems Biomedical Science, Soongsil University, Seoul 06978, Republic of Korea; ^4^Department of Integrative Biotechnology, Sungkyunkwan University, Suwon 16419, Republic of Korea

## Abstract

Despite a large number of studies reporting a variety of biological and pharmacological activities of* Momordica charantia*, its skin protective properties are poorly understood. The present study aimed to explore the skin protective properties of* Momordica charantia* methanol extract (Mc-ME) and the underlying mechanism in keratinocytes, fibroblasts, and melanocytes. Mc-ME exhibited an antioxidative property by decreasing radical levels in HaCaT keratinocytes and a cytoprotective property in H_2_O_2_-damaged HaCaT cells, which was mediated by increasing the expression or activation of Kelch-like ECH-associated protein 1 (KEAP1), HO-1, p85/PI3K, and AKT. Mc-ME was also active against wrinkle formation by regulating the activity or expression of tissue remodeling factors such as elastase, type 1 collagen, and matrix metalloproteinase (MMP)-1 and -9 and tissue-protecting enzymes such as hemeoxygenase-1 (HO-1) and sirtuin 1 (SIRT1) in NIH3T3 fibroblasts and HaCaT cells, in addition to increasing the proliferation of HaCaT cells. Mc-ME also showed antidehydration properties by inducing the expression of natural moisturizing factors such as filaggrin (FLG), transglutaminase-1 (TGM-1), and hyaluronic acid synthase (HAS)-1, -2, and -3 in HaCaT cells. Moreover, Mc-ME showed an antimelanogenic property by inhibiting the synthesis and secretion of melanin from B16F10 melanoma cells via suppression of tyrosinase activity. Taken together, these results suggest that Mc-ME plays a skin protective role through its antioxidative, cytoprotective, skin remodeling, moisturizing, and antimelanogenic properties and might be a new and promising skin protective cosmeceutical.

## 1. Introduction

Skin is the outer thin layer of tissue covering the animal body that protects the body from environmental stresses and dangers, including infectious pathogens, ultraviolet (UV) light, harmful chemical agents, and mechanical stimulation, in addition to maintaining body temperature and moisture. Chronic and repeated exposure of skin to these stresses causes detrimental damage, resulting in ageing and cancers of skin [1–3]. Skin damage involves various physiological changes in skin tissues. UV and oxidative stress induce inflammation and oxidation in skin tissues by generating nitric oxide (NO) and free radicals such as reactive oxygen/nitrogen species (ROS/RNS) [4–7]. Increased inflammatory responses and oxidation facilitate apoptotic cell death [8], which is considered one of the major causative factors of ageing and ageing-associated diseases [9, 10]. UV also induces skin tissue remodeling and wrinkle generation by modulating the expression of tissue remodeling factors such as procollagen, matrix metalloproteinases (MMPs), and elastase [11–13]. Skin ageing induces dehydration in skin tissues, and hyaluronic acid (HA) has been reported as a key molecule involved in skin hydration through regulating the expression of hyaluronic acid synthases (HASs) [14]. In addition, filaggrin (FLG) and transglutaminase-1 (TGM-1) have been reported as natural moisturizing factors with moisturization and skin barrier functions [5, 15]. Melanin is a dark pigment that is synthesized in melanocytes by the oxidation of L-tyrosine and protects the skin from external stimuli such as UV [16, 17]. Although the primary role of melanin is to protect skin tissues from UV irradiation, excessive production of melanin causes the generation of age spots and freckles, and many efforts have been made to develop preparations that reduce melanin synthesis for use as whitening constituents [5, 18–20].

*Momordica charantia*, also known as a bitter melon or African cucumber, is a vine belonging to the Cucurbitaceae family. It is widely found in tropical and subtropical regions of the world, such as Caribbean, Asian, and East African countries, and is used in traditional medicines [21, 22]. Various active biological components have been identified, including charantin, vicine, mormordin, flavonoids, polyphenols, carotenoids, vitamin C, and plant insulin [23].* Momordica charantia* has been reported to have various biological functions, including anti-inflammatory, immunostimulatory, antioxidative, antibacterial, antiviral, antifungal, antidiabetic, cardioprotective, hypoglycemic, hypocholesterolemic, and antitumor activities [22, 24–27]. However, there are limited studies on the skin-protective activity of* Momordica charantia* and the underlying mechanism. Therefore, the present study explored the skin-protective effect of* Momordica charantia* methanol extract (Mc-ME) by examining its antioxidative, cytoprotective, skin tissue remodeling, moisturizing, and antipigmentation activities in HaCaT keratinocytes, NIH3T3 fibroblasts, and B16F10 melanocytes. Moreover, the mechanism of Mc-ME–mediated skin protective activity was further investigated in these cells.

## 2. Materials and Methods

### 2.1. Materials

HaCaT, NIH3T3, and B16F10 cells were purchased from* American Type Culture Collection* (Rockville, MD, USA). Dulbecco's Modified Eagle's medium (DMEM), fetal bovine serum (FBS), phosphate-buffered saline (PBS), streptomycin, penicillin, and L-glutamine were purchased from Gibco (Grand Island, NY, USA). 2,2-Diphenyl-1-picrylhydrazyl (DPPH), sodium nitroprusside (SNP), 2′,7′-dichlorofluorescin diacetate (H_2_DCFDA), ascorbic acid, 3-(4,5-dimethylthiazol-2-yl)-2,5-diphenyltetrazolium bromide (MTT), sodium dodecyl sulfate (SDS), hydrogen peroxide (H_2_O_2_),* 3β-hydroxy-12-ursen-28-ic acid* (ursolic acid), N-succinyl-Ala-Ala-Ala-p-nitroanilide (STANA), phorbol-12-myristate-13-acetate (PMA), retinol (RE), epidermal growth factor (EGF), LY294002, L-3,4-dihydroxyphenylalanine (L-DOPA), 5-hydroxy-2-hydroxymethyl-4H-pyranone (kojic acid), monophenol monooxygenase (mushroom tyrosinase), 4-hydroxyphenyl-*β*-D-glucopyranoside (arbutin), and *α*-melanocyte stimulating hormone (*α*-MSH) were purchased from Sigma Chemical Co. (St. Louis, MO, USA). TRI reagent® was purchased from Molecular Research Center Inc. (Cincinnati, OH, USA). MuLV reverse transcriptase was purchased from ThermoFisher Scientific (Waltham, MA, USA). Primers specific for matrix metalloproteinase (MMP)-1, MMP-9, hemeoxygenase-1 (HO-1), type 1 pro-collagen (COL1A1), filaggrin (FLG), transglutaminase-1 (TGM-1), hyaluronic acid synthase (HAS)-1, HAS-2, HAS-3, and GAPDH for semiquantitative reverse transcriptase-polymerase chain reaction (RT-PCR) were synthesized at Bioneer Inc. (Daejeon, Korea). Antibodies specific for total and phospho-forms of Kelch-like ECH-associated protein 1 (KEAP1), HO-1, p85/PI3K, Src, AKT, and *β*-actin were purchased from Cell Signaling Technology (Beverly, MA, USA) or Santa Cruz Biotechnology (Santa Cruz, CA, USA). Enhanced chemiluminescence reagents were purchased from AbFrontier (Seoul, Korea).

### 2.2. Preparation of Mc-ME

*M. charantia* methanol extract (Mc-ME, Code No.: 034-065) was obtained from the Korean Plant Extract Bank (KPEB) in the Plant Diversity Research Center (http://extract.kribb.re.kr/, e-mail: plantext@kribb.re.kr Daejeon, Korea). Briefly, dried whole plant (100 g) of* Momordica charantia*, collected at Cheolwon area (longitude: 127.313 and latitude: 38.146), Korea, in 2008, was pulverized to powder using a mechanical grinder after dried at 60°C for 24 h, passed through a 60-mesh sieve, and extracted with 95% methanol (1 l x 3) for 48 h in the soxhlet apparatus. The extract was then filtered with filter paper, concentrated under reduced pressure in rotary evaporator, and finally dried in Vacuum Freeze Dryer Clean-Vac 12 (Biotron, Gangneung, Korea). The yield of the extract was approximately 12.9%. The crude extract was stored in 4°C to use in the experiment.

### 2.3. Cell Culture

HaCaT, a human keratinocyte cell line; NIH3T3, a murine embryo fibroblast cell line; and B16F10, a murine melanoma cell line, were cultured in DMEM supplemented with 10% FBS, streptomycin (100 mg/ml), penicillin (100 U/ml), and L-glutamine (2 mM) at 37°C in a 5% CO_2_ humidified incubator.

### 2.4. MTT Assay

Cell viability was determined by a conventional MTT assay as previously reported [28]. Briefly, to test Mc-ME cytotoxicity, HaCaT, NIH3T3, and B16F10 cells were treated with Mc-ME (0–400 *μ*g/ml) for 24 h. To test the antioxidative activity of Mc-ME (0–200 *μ*g/ml), HaCaT cells were treated with Mc-ME in the absence or presence of H_2_O_2_ (50 *μ*M) and LY294002 (20 *μ*M) for 24 h. To test the cytoprotective activity of Mc-ME, HaCaT cells pretreated with Mc-ME (0–200 *μ*g/ml) for 30 min were treated with H_2_O_2_ (50 *μ*M) for 24 h.

### 2.5. Radical Scavenging Assay

Radical scavenging activity of Mc-ME was determined by a DPPH assay as previously reported [29]. DPPH (150 *μ*M) was mixed with either Mc-ME (0–200 *μ*g/ml) or ascorbic acid (100 *μ*M) and incubated for 20 min at room temperature. Absorbance was determined at 517 nm using a Spectra Max 250 microplate reader (Molecular Devices, Sunnyvale, CA, USA).

### 2.6. ROS Generation Assay

The effect of Mc-ME on ROS generation was determined by a H_2_DCFDA assay. HaCaT cells were pretreated with Mc-ME (0–200 *μ*g/ml) for 30 min and then treated with SNP (200 *μ*M) for 24 h, followed by incubation with H_2_DCFDA (10 *μ*M) at 37°C for 20 min. The cells were washed with PBS three times, and fluorescence was determined using a flow cytometer (EMD Millipore Co., Billerica, MA, USA).

### 2.7. Elastase Inhibitory Assay

Elastase inhibitory activity of Mc-ME was carried out according to previous report [30]. Briefly, elastase (0.3 units/ml) and the substrate STANA (5 mM) were mixed with either Mc-ME (0–400 *μ*g/ml) or ursolic acid (50 *μ*M) and incubated for 15 min at room temperature. NIH3T3 cells in the absence or presence of Mc-ME (0–200 *μ*g/ml) were irradiated at 30 mJ/cm^2^ for 10 sec using a UVB lamp (Bio-Link BLX-312, VILBER LOURMAT, France) and further incubated for 24 h. Cell lysate was mixed with elastase (0.3 units/ml) and STANA (5 mM) and incubated for 15 min at room temperature. The absorbance was determined at 410 nm using a Spectra Max 250 microplate reader.

### 2.8. Semiquantitative RT-PCR

HaCaT cells were pretreated with Mc-ME (0–200 *μ*g/ml) for 30 min and then treated with either H_2_O_2_ (50 *μ*M) or PMA (100 nM) for 6 h. HaCaT cells were also treated with either Mc-ME (0–200 *μ*g/ml) or RE (10 *μ*g/ml) for 6 h. Total RNA was extracted from these cells using TRI reagent® according to the manufacturer's instructions and stored at -70°C until use. cDNA was synthesized from total RNA (1 *μ*g) using MuLV reverse transcriptase according to the manufacturer's instructions. Semiquantitative RT-PCR was conducted as previously described [5, 12], and the sequences of the primers used in this study are listed in Table 1.

### 2.9. Cell Proliferation Assay

HaCaT cells were treated with either Mc-ME (0–200 *μ*g/ml), RE (5 *μ*g/ml), or EGF (1 ng/ml) for 72 h, and the cell numbers were counted every 24 h using a hemocytometer.

### 2.10. Western Blot Analysis

HaCaT cells were pretreated with Mc-ME (0–200 *μ*g/ml) for 30 min and then treated with H_2_O_2_ (50 *μ*M) for 24 h. For western blot analysis, total cell lysates were prepared as previously described [31], subjected to SDS-polyacrylamide gel electrophoresis, and transferred to polyvinylidene fluoride membranes. Total and phosphorylated forms of target proteins were detected using specific antibodies and visualized using enhanced chemiluminescence reagents.

### 2.11. Tyrosinase Activity Assay

B16F10 cells were treated with either Mc-ME (0–400 *μ*g/ml) or kojic acid (300 *μ*M) for 24 h, and total cell lysates were incubated with mushroom tyrosinase (100 units/ml) and L-DOPA (1 mg/ml) for 15 min at room temperature. Tyrosinase activity was determined by measuring the absorbance of the mixture at 475 mm using a Spectra Max 250 microplate reader.

### 2.12. Melanin Generation Assays

B16F10 cells were treated with *α*-MSH (100 nM) and either Mc-ME (0–400 *μ*g/ml) or arbutin (1 mM) for 48 h. To determine melanin content in the cells, the cells were lysed using lysis buffer (50 mM Tris-HCl pH 7.5, 20 mM NaF, 25 mM *β*-glycerophosphate pH 7.5, 120 mM NaCl, and 2% NP-40), followed by centrifugation (13,000 × g for 10 min). The cell pellets were resuspended in 10% DMSO in 1 N NaOH and incubated for 30 min at 50°C. The final cell pellets were collected by centrifugation (13,000 × g for 10 min), and the melanin content was determined by measuring the absorbance at 405 nm using a Spectra Max 250 microplate reader. To determine melanin secretion from the cells, the absorbance of the cell culture media was measured at 475 nm using a Spectra Max 250 microplate reader.

### 2.13. Statistical Analysis

All data are presented as a mean ± standard deviation (SD) of at least three independent experiments. A Mann-Whitney test was used to compare statistical differences between experimental and control groups. A* P* value < 0.05 was considered statistically significant (*∗P* < 0.05, *∗∗P* < 0.01). All statistical analyses were conducted using the SPSS program (SPSS Inc., Chicago, IL, USA).

## 3. Results

### 3.1. Antioxidative and Cytoprotective Effects of Mc-ME in HaCaT Cells

**Figure 1:**
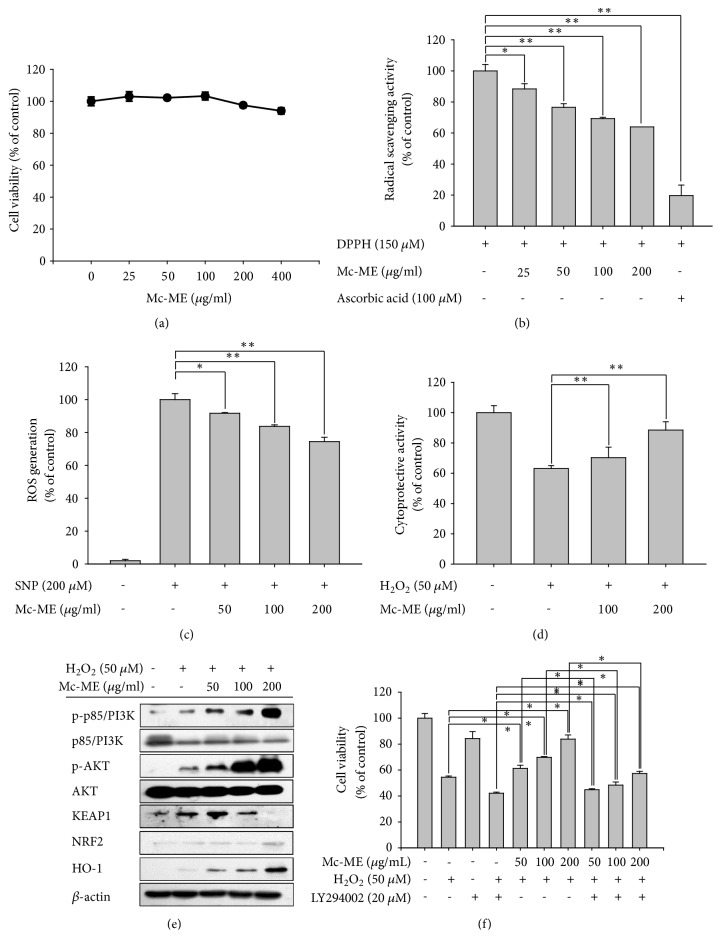
*Antioxidative and cytoprotective effects of Mc-ME in HaCaT cells*. (a) HaCaT cells were treated with increasing doses of Mc-ME (0–400 *μ*g/ml) for 24 h, and cell viability was determined by a conventional MTT assay. (b) DPPH (150 *μ*M) was mixed with either increasing doses of Mc-ME (0–200) or ascorbic acid (100 *μ*M) and incubated for 20 min. The radical scavenging activity was determined by measuring the absorbance at 517 nm. (c) HaCaT cells were pretreated with increasing doses of Mc-ME (0–200 *μ*g/ml) for 30 min and then treated with SNP (200 *μ*M) for 24 h. The cells were incubated with H_2_DCFDA (10 *μ*M) at 37°C for 20 min, and ROS levels were determined by measuring fluorescence using a flow cytometer. (d) HaCaT cells were pretreated with increasing doses of Mc-ME (0–200 *μ*g/ml) and then treated with H_2_O_2_ (50 mM) for 24 h. Cell viability was determined by a conventional MTT assay. (e) HaCaT cells were pretreated with increasing doses of Mc-ME (0–200 *μ*g/ml) and then treated with H_2_O_2_ (50 *μ*M) for 24 h. Levels of total and phosphorylated KEAP1, HO-1, p85/PI3K, and AKT in the total cell lysates were determined by western blot analysis. (f) HaCaT cells were treated with H_2_O_2_ (50 mM) and/or LY294002 (20 *μ*M) in the absence or presence of increasing doses of Mc-ME (0–200 *μ*g/ml) for 24 h and cell viability was determined by a conventional MTT assay. *∗P* < 0.05, *∗∗P* < 0.01 compared to control.

Antioxidative and cytoprotective effects of Mc-ME on skin were investigated in HaCaT keratinocytes. First, the cytotoxicity of Mc-ME was evaluated by treating HaCaT cells with increasing doses of Mc-ME (0–400 *μ*g/ml) for 24 h. Results of the MTT assay demonstrated that these concentrations of Mc-ME did not show significant cytotoxicity in HaCaT cells (Figure 1(a)). Next, the antioxidative effect of Mc-ME was examined by measuring the radical scavenging activity and inhibition of ROS generation in HaCaT cells. DPPH radicals (150 *μ*M) were incubated with either increasing doses of Mc-ME (0–200 *μ*g/ml) or ascorbic acid (100 *μ*M), as a positive control, for 20 min. Mc-ME significantly reduced DPPH radical level in a dose-dependent manner (Figure 1(b)). In addition, HaCaT cells were pretreated with increasing doses of Mc-ME (0–200 *μ*g/ml) for 30 min and then treated with SNP (200 *μ*M) for 24 h, and ROS generation induced by SNP in the cells was decreased by Mc-ME in a dose-dependent manner (Figure 1(c)). The protective effect of Mc-ME on oxidative stress-induced cytotoxicity of skin cells was further examined. HaCaT cells were pretreated with increasing doses of Mc-ME (0–200 *μ*g/ml) and then treated with H_2_O_2_ (50 *μ*M) for 24 h. H_2_O_2_–induced cytotoxicity of the HaCaT cells was reduced by Mc-ME in a dose-dependent manner (Figure 1(d)). To examine the molecular mechanism of the Mc-ME–mediated cytoprotective effect in H_2_O_2_–treated HaCaT cells, the activities of p85/PI3K and AKT were evaluated by western blot analysis. Mc-ME increased the phosphorylation of p85/PI3K and AKT in H_2_O_2_–treated HaCaT cells in a dose-dependent manner (Figure 1(e)). In addition, Mc-ME increased protein expression of KEAP1 (50 *μ*g/ml Mc-ME) and HO-1 (50–200 *μ*g/ml Mc-ME) in H_2_O_2_–treated HaCaT cells (Figure 1(e)). LY294002, a selective p85/PI3K inhibitor, suppressed the Mc-ME–mediated cytoprotective effect on H_2_O_2_–treated HaCaT cells in a dose-dependent manner (Figure 1(f)), confirming the role of p85/PI3K in the Mc-ME–mediated cytoprotective effect.

### 3.2. Regulatory Effect of Mc-ME on Skin Tissue Remodeling Factors in NIH3T3 and HaCaT Cells

**Figure 2:**
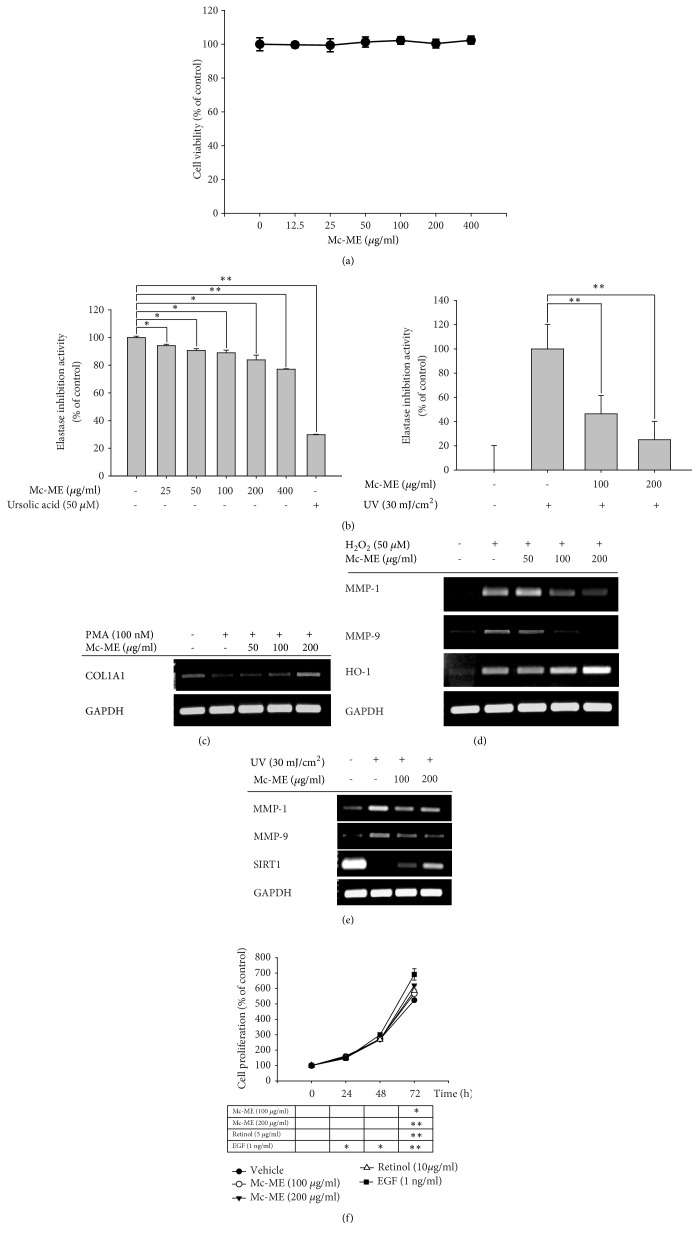
*Regulatory effect of Mc-ME on skin tissue remodeling factors in NIH3T3 and HaCaT cells*. (a) NIH3T3 cells were treated with increasing doses of Mc-ME (0–400 *μ*g/ml) for 24 h, and the cell viability was determined by a conventional MTT assay. ((b); left panel) Elastase (0.3 units/ml) and STANA (5 mM) were mixed with either increasing doses of Mc-ME (0–400 *μ*g/ml) or ursolic acid (50 *μ*M), followed by incubation for 15 min. ((b); right panel) NIH3T3 cells were irradiated at 30 mJ/cm^2^ for 10 sec in the absence or presence of increasing doses of Mc-ME (0–200 *μ*g/ml) followed by incubation for 24 h. Cell lysates were incubated with elastase (0.3 units/ml) and STANA (5 mM) for 15 min. Elastase activity was determined by measuring the absorbance at 410 nm. (c) HaCaT cells were pretreated with increasing doses of Mc-ME (0–200 *μ*g/ml) for 30 min and then treated with PMA (100 nM) for 24 h. mRNA expression level of COL1A1 was determined by semiquantitative RT-PCR. (d) HaCaT cells were pretreated with increasing doses of Mc-ME (0–200 *μ*g/ml) for 30 min and then treated with H_2_O_2_ (50 *μ*M) for 24 h. mRNA expression levels of MMP-1, -9, and HO-1 were determined by semiquantitative RT-PCR. (e) HaCaT cells were irradiated at 30 mJ/cm^2^ for 10 sec in the absence or presence of increasing doses of Mc-ME (0–200 *μ*g/ml), and mRNA expression levels of MMP-1, -9, and SIRT1 were determined by semiquantitative RT-PCR. (f) HaCaT cells were treated with increasing doses of Mc-ME (0–200 *μ*g/ml), RE (10 *μ*g/ml), or EGF (1ng/ml) for 72 h, and the cell proliferation level was determined by measuring cell viability with a conventional MTT assay every 24 h. *∗P* < 0.05, *∗∗P* < 0.01 compared to control.

The effect of Mc-ME on the factors responsible for skin tissue remodeling was investigated in NIH3T3 fibroblasts. First, the cytotoxicity of Mc-ME was evaluated by treating NIH3T3 cells with increasing doses of Mc-ME (0–400 *μ*g/ml) for 24 h, and no cytotoxicity was observed for all concentrations of Mc-ME (Figure 2(a)). To examine the effect of Mc-ME on elastase activity, elastase was mixed with its substrate and either increasing doses of Mc-ME (0–400 *μ*g/ml) or ursolic acid (50 *μ*M), as a positive control, and elastase activity was measured. Mc-ME inhibited elastase activity in a dose-dependent manner (Figure 2(b); left panel). The effect of Mc-ME on elastase activity was further examined in NIH3T3 cells irradiated with UV in the presence or absence of Mc-ME (0–200 *μ*g/ml). Mc-ME significantly inhibited elastase activity in a dose-dependent manner (Figure 2(b); right panel). The effect of Mc-ME on mRNA expression of COL1A1, MMPs, HO-1, and SIRT1 in HaCaT cells was examined by semiquantitative RT-PCR. Mc-ME (0–200 *μ*g/ml) increased the mRNA expression level of COL1A1 that was decreased by PMA in a dose-dependent manner (Figure 2(c)). Mc-ME (0–200 *μ*g/ml) also increased the mRNA expression level of HO-1 and decreased the mRNA expression levels of MMP-1 and MMP-9 that were increased by H_2_O_2_ in HaCaT cells in a dose-dependent manner (Figure 2(d)). Similarly, Mc-ME (0–200 *μ*g/ml) dose-dependently decreased mRNA expression levels of MMP-1 and MMP-9 that were increased by UV irradiation (Figure 2(e)) and increased mRNA expression level of SIRT1 that was decreased by UV irradiation (Figure 2(e)) in HaCaT cells. Finally, the effect of Mc-ME on cell proliferation was examined by treating HaCaT cells with either increasing doses of Mc-ME (0–200 *μ*g/ml) or EGF, a positive control, for 72. Mc-ME at both 100 and 200 *μ*g/ml induced HaCaT cell proliferation at 72 h (Figure 2(f)).

### 3.3. Regulatory Effect of Mc-ME on Moisturizing Factors in HaCaT Cells

**Figure 3:**
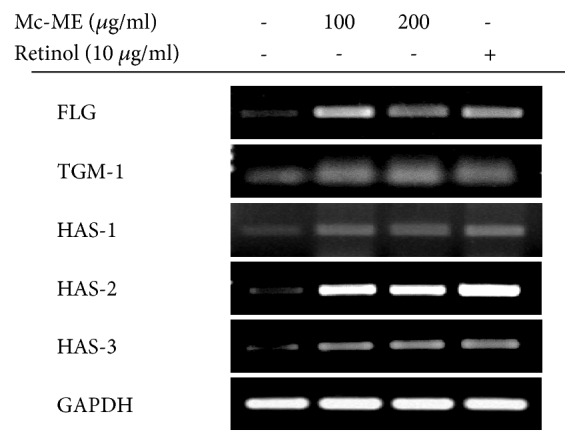
*Regulatory effect of Mc-ME on moisturizing factors in HaCaT cells*. (a) HaCaT cells were treated with either increasing doses of Mc-ME (0–200 *μ*g/ml) or RE (10 *μ*g/ml) for 24 h, and mRNA expression levels of FLG, TGM-1, HAS-1, -2, and -3 were determined by semiquantitative RT-PCR.

To examine the effect of Mc-ME on mRNA expression levels of moisturizing factors, HaCaT cells were treated with either increasing doses of Mc-ME (0–200 *μ*g/ml) or RE (10 *μ*g/ml), as a positive control, and mRNA expression levels of moisturizing factors such as FLG, TGM-1, HAS-1, HAS-2, and HAS-3 were determined by semiquantitative RT-PCR. Similar to the effects of RE, Mc-ME upregulated mRNA expression of FLG, TGM-1, HAS-1, HAS-2, and HAS-3 genes in HaCaT cells at both 100 and 200 *μ*g/ml (Figure 3).

### 3.4. Antimelanogenic Effect of Mc-ME in B16F10 Cells

**Figure 4:**
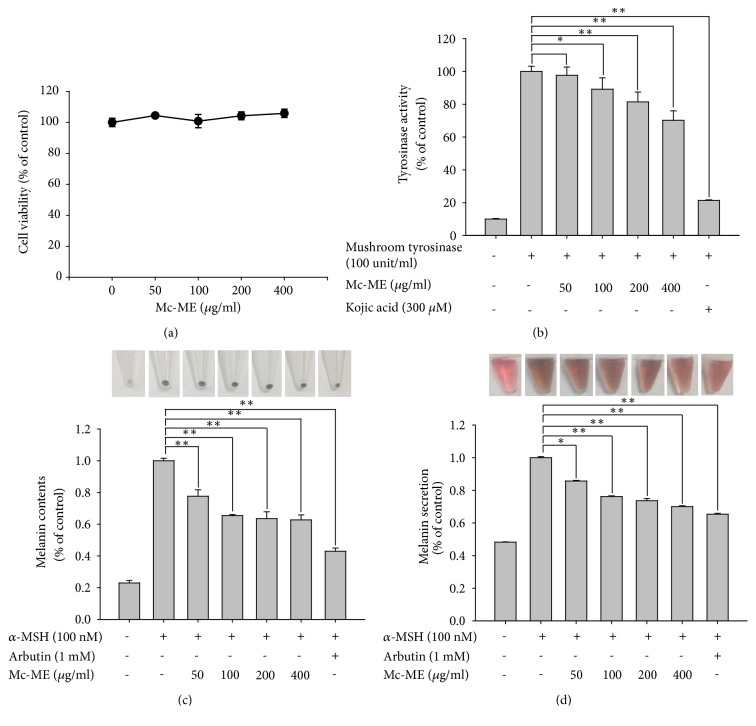
*Antimelanogenic effect of Mc-ME in B16F10 cells*. (a) B16F10 cells were treated with increasing doses of Mc-ME (0–400 *μ*g/ml) for 24 h, and cell viability was determined by a conventional MTT assay. (b) Total lysates of B16F10 cells treated with either increasing doses of Mc-ME (0–400 *μ*g/ml) or kojic acid (300 *μ*M) for 24 h were incubated with mushroom tyrosinase (100 units/ml) and L-DOPA (1 mg/ml) for 15 min, and tyrosinase activity was determined by measuring the absorbance at 475 nm. (c) B16F10 cells were pretreated with either increasing doses of Mc-ME (0–400 *μ*g/ml) or arbutin (1 mM) for 30 min and then treated with *α*-MSH (100 nM) for 48 h. The melanin content in the cells was imaged by photography and determined quantitatively by measuring the absorbance at 405 nm. (d) B16F10 cells were pretreated with either increasing doses of Mc-ME (0–400 *μ*g/ml) or arbutin (1 mM) for 30 min and then treated with *α*-MSH (100 nM) for 48 h. The level of melanin secreted from the cells into culture media was assessed by photography to compare the color of the medium and determined quantitatively by measuring the absorbance at 475 nm. *∗P* < 0.05, *∗∗P* < 0.01 compared to control.

The effect of Mc-ME on melanogenesis was investigated in B16F10 melanoma cells stimulated with *α*-MSH. First, the cytotoxicity of Mc-ME was evaluated by treating B16F10 cells with increasing doses of Mc-ME (0–400 *μ*g/ml) for 24 h, and no cytotoxicity was observed at all concentrations (Figure 4(a)). To examine the effect of Mc-ME on tyrosinase activity, lysates of B16F10 cells treated with either increasing doses of Mc-ME (0–400 *μ*g/ml) or kojic acid (300 *μ*M) as a positive control were incubated with mushroom tyrosinase and its substrate, L-DOPA, and tyrosinase activity was determined. Mc-ME suppressed tyrosinase activity in a dose-dependent manner (Figure 4(b)). Moreover, Mc-ME significantly reduced melanin content (Figure 4(c)) and melanin secretion (Figure 4(d)) induced by *α*-MSH in B16F10 cells.

## 4. Discussion

*Momordica charantia* (Cucurbitaceae) is widely grown and has long been used in traditional medicines in tropical and subtropical countries [21, 22]. Moreover, a large number of studies have demonstrated that* Momordica charantia* has anti-inflammatory, immunostimulatory, antioxidative, antibacterial, antiviral, antifungal, antidiabetic, cardioprotective, hypoglycemic, hypocholesterolemic, and antitumor activities and can ameliorate the symptoms of various human diseases [22, 24–27]. In spite of studies reporting a variety of biological and pharmacological activities of* Momordica charantia*, there are few reports of the protective effects of* Momordica charantia *on skin. Therefore, the present study aimed to explore the skin protective activity of* Momordica charantia* and the underlying mechanism using keratinocytes, fibroblasts, and melanocytes under conditions of stimulation to induce skin damage.

Oxidative stress is one of the critical causative factors of skin damage [32]; therefore, we first evaluated the antioxidative effect of Mc-ME in HaCaT keratinocytes. For many agents, cytotoxicity is one of the major hurdles for drug development despite a good pharmacological effect. We first demonstrated that Mc-ME did not show significant cytotoxicity in HaCaT cells (Figure 1(a)). Next, we evaluated the antioxidative effect of Mc-ME by measuring its radical scavenging and ROS inhibitory activities. Mc-ME not only significantly induced radical scavenging (Figure 1(b)), but also inhibited ROS generation in HaCaT cells stimulated by the oxidative inducer SNP (Figure 1(c)) in a dose-dependent manner, indicating that Mc-ME may contribute to prevention of skin damage caused by oxidative stress inducers.

Oxidative stress markedly induces severe damage of cellular structures, resulting in permanent cell death [32], implying that inhibition of skin cell death is a protective mechanism to prevent skin tissues from detrimental risk factors such as an oxidative stress. We showed that Mc-ME exhibited cytoprotective effects in H_2_O_2_–induced HaCaT cells in a dose-dependent manner (Figure 1(d)). We further investigated the molecular mechanism by which Mc-ME plays an antioxidative and cytoprotective role in keratinocytes under oxidative stress and showed that Mc-ME increased the expression of antioxidative proteins such as KEAP1 [33] and HO-1 [34] in the H_2_O_2_–induced HaCaT cells (Figure 1(e)). p85/PI3K and AKT, downstream molecules of p85/PI3K, are critical intracellular signaling proteins in cell survival and proliferation [35, 36], and Mc-ME dramatically induced activation of p85/PI3K and AKT in H_2_O_2_–induced HaCaT cells (Figure 1(e)). Accordingly, Mc-ME increased the viability of H_2_O_2_–induced HaCaT cells in a dose-dependent manner, and this effect was significantly decreased by inhibition of p85/PI3K using its selective inhibitor, LY294002 (Figure 1(f)). These results suggest that Mc-ME plays cytoprotective and skin regenerative roles in oxidative stress–induced skin cells by increasing the expression of antioxidative proteins and by activating the p85/PI3K-AKT signaling pathway that is critical for cell survival and proliferation.

A wrinkle is a fold or crease in the skin, and formation of skin wrinkles is one symptom of the ageing process. Several factors are involved in wrinkle formation during the ageing process, including genetics, hormonal alteration, and external stresses such as UV and inflammatory or oxidative inducers [37]. Regarding the cellular and molecular aspects of wrinkle formation, reduction in keratinocytes and fibroblasts in the extracellular matrix is a major cause. In addition, regulation of the expression or activities of extracellular matrix proteins such as collagens, MMPs, and elastases is strongly associated with the loss of extracellular matrix protein in skin tissues, resulting in wrinkle formation [38]. Therefore, we next investigated the effect of Mc-ME on the activity and expression of extracellular matrix proteins in NIH3T3 fibroblasts. First, we confirmed that Mc-ME exerted no cytotoxicity in NIH3T3 cells (Figure 2(a)). Mc-ME decreased elastase activity (Figure 2(b); left panel) and reduced the UV irradiation–induced increase in elastase activity in NIH3T3 cells (Figure 2(b); right panel). Mc-ME also increased mRNA expression of type 1 collagen that was reduced by the inflammation inducer PMA (Figure 2(c)), and decreased mRNA expression of MMP-1 and -9 that was induced by the oxidative stress H_2_O_2_ (Figure 2(d)) in HaCaT cells. As expected, mRNA expression of the antioxidative protein HO-1 [34] was increased by Mc-ME in HaCaT cells (Figure 2(d)). Moreover, Mc-ME reduced mRNA expression of MMP-1 and -9 that was induced by UV irradiation and induced mRNA expression of SIRT1, which was reported to attenuate tissue atrophy [39], that was suppressed by UV irradiation in HaCaT cells (Figure 2(e)). These results strongly suggest that Mc-ME may prevent wrinkle formation through its activities in fibroblasts and keratinocytes by suppressing and downregulating the activity and expression of elastases and MMPs that induce wrinkle formation as well as upregulating the expression of collagen, HO-1, and SIRT1 that prevent wrinkle formation in skin tissues. Since impaired proliferation of skin cells is one of the main causes of wrinkle formation during the ageing process, we also evaluated the effect of Mc-ME on the proliferation of keratinocytes and showed that Mc-ME increased the proliferation of HaCaT cells (Figure 2(f)). This result indicates that, together with preventing degradation of the extracellular matrix, Mc-ME may suppress wrinkle formation by inducing the proliferation of skin cells.

Dehydration is another detrimental factor that damages skin tissues during the ageing process. We investigated the effect of Mc-ME on moisturization of skin tissues and showed that Mc-ME induced mRNA expression of natural moisturizing factors, such as FLG, TGM-1 [5, 15], and HAS family members, including HAS-1, -2, and -3 [14], in HaCaT keratinocytes (Figure 3). It was reported that RE induces the expression of natural moisturizing factors, HAS family members, and HA in human skin tissues [40]. Interestingly, the effect of Mc-ME on the expression of FLG, TGM-1, HAS-1, -2, and -3 was comparable to that of RE (Figure 3), suggesting that Mc-ME might be a new and promising antidehydrating agent for skin tissues during the process of ageing.

In spite of the skin-protective role of melanin against UV irradiation, hyperproduction of melanin causes the formation of age spots and freckles, which has driven a number of studies to develop preparations that suppress melanin generation for use as whitening constituents [5, 18–20]. Therefore, we finally investigated the effect of Mc-ME on the melanin-induced pigmentation of skin tissues using melanoma cells. Like keratinocytes (Figure 1(a)) and fibroblasts (Figure 2(a)), Mc-ME did not exhibit cytotoxicity in B16F10 melanoma cells (Figure 4(a)). As tyrosinase is a critical determinant of melanin synthesis in melanocytes [41], we examined the effect of Mc-ME on tyrosinase activity and showed that Mc-ME dose-dependently suppressed tyrosinase activity in B16F10 cells (Figure 4(b)). We next examined the effect of Mc-ME on the production of melanin from melanocytes by measuring melanin content and secretion in B16F10 cells stimulated with *α*-MSH, which induces melanogenesis by activating melanogenesis-specific transcription factor and enzymes associated with melanin synthesis [42, 43]. As expected, Mc-ME effectively suppressed the production and secretion of melanin from melanocytes induced by *α*-MSH (Figures 4(c) and 4(d)). These results indicate that Mc-ME suppresses the production and secretion of melanin from melanocytes by inhibiting the activity of tyrosinase, a critical enzyme for melanogenesis in melanocytes.

**Figure 5:**
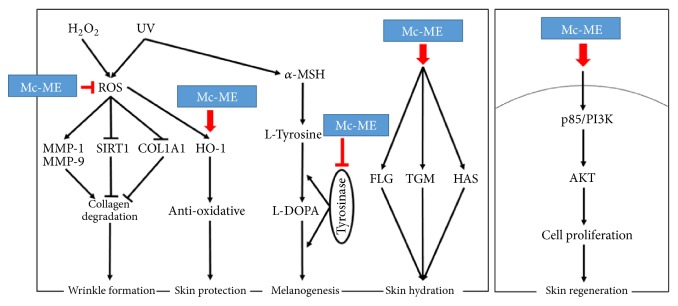
Schematic summary demonstrating the pathways of Mc-ME-mediated skin-protective property.

In conclusion, we demonstrated a skin-protective activity of Mc-ME and elucidated the underlying mechanisms under various conditions that induce skin damage, such as oxidative stress, UV irradiation, inflammatory stress, and melanogenic inducers. Our data showed that the skin-protective property of Mc-ME was mediated through various activities. First, Mc-ME exhibited antioxidative and cytoprotective activities in keratinocytes via increased expression of antioxidative proteins such as KEAP1 and HO-1 and activation of intracellular kinases responsible for cell proliferation such as p85/PI3K and AKT. Second, Mc-ME exhibited antiwrinkle formation activity by not only regulating the activity and expression of skin remodeling factors and the proteins responsible for reducing oxidative stress and tissue atrophy, but also inducing cell proliferation in fibroblasts and keratinocytes. Third, Mc-ME showed moisturizing activity through increased expression of natural moisturizing factors such as FLG, TGM-1, and HAS family members in keratinocytes. Finally, Mc-ME suppressed melanin generation and secretion from melanocytes by inhibiting the activity of tyrosinase, a critical enzyme in the induction of melanogenesis in melanocytes. These skin-protective activities of Mc-ME in keratinocytes, fibroblasts, and melanocytes are summarized in Figure 5, as shown in the case of treatment with* Artemisia asiatica* ethanol extract [44]. Taken together, these results strongly indicate that Mc-ME plays a protective role against various skin damaging stresses and suggest that Mc-ME could have potential applications in cosmeceutical preparations.

## Figures and Tables

**Table 1:** Sequences of primers used in semiquantitative RT-PCR.

Name	Primer	Sequence (5′ to 3′)
MMP-1	Forward	TCTGACGTTGATCCCAGAGAGCAG
	Reverse	CAGGGTGACACCAGTGACTGCAC
MMP-9	Forward	GCCACTTGTCGGCGATAAGG
	Reverse	CACTGTCCACCCCTCAGAGC
HO-1	Forward	TGAAGGAGGCCACCAAGGAGG
	Reverse	AGAGGTCACCCAGGTAGCGGG
COL1A1	Forward	AGGGCCAAGACGAAGACATC
	Reverse	AGATCACGTCATCGCACAACA
FLG	Forward	AGGGAAGATCCAAGAGCCCA
	Reverse	ACTCTGGATCCCCTACGCTT
TGM-1	Forward	AGGGAAGATCCAAGAGCCCA
	Reverse	ACTCTGGATCCCCTACGCTT
HAS-1	Forward	GAAATGCGGCAGATGACGAC
	Reverse	AACTCCCCAGCGTCTGATTG
HAS-2	Forward	CCACCCAGTACAGCGTCAAC
	Reverse	CATGGTGCTTCTGTCGCTCT
HAS-3	Forward	TTCTTTATGTGACTCATCTGTCTCACCGG
	Reverse	ATTGTTGGCTACCAGTTTATCCAAACG
SIRT1	Forward	TCGCAACTATACCCAGAACATAGACA
	Reverse	CTGTTGCAAAGGAACCATGACA
GAPDH	Forward	CACTCACGGCAAATTCAACGGCAC
	Reverse	GACTCCACGACATACTCAGCAC

## Data Availability

The data used to support the findings of this study are available from the corresponding author upon request.
